# GRPEL2 Knockdown Exerts Redox Regulation in Glioblastoma

**DOI:** 10.3390/ijms222312705

**Published:** 2021-11-24

**Authors:** Chi-Tun Tang, Yao-Feng Li, Chung-Hsing Chou, Li-Chun Huang, Shih-Ming Huang, Dueng-Yuan Hueng, Chia-Kuang Tsai, Yuan-Hao Chen

**Affiliations:** 1 Department of Neurological Surgery, Tri-Service General Hospital, National Defense Medical Center, Taipei 11490, Taiwan; 803010304@mail.ndmctsgh.edu.tw (C.-T.T.); hondy2195@yahoo.com.tw (D.-Y.H.); 2 Graduate Institute of Medical Sciences, National Defense Medical Center, Taipei 11490, Taiwan; choutpe@yahoo.com.tw (C.-H.C.); shihming7102@gmail.com (S.-M.H.); 3 Department of Pathology, Tri-Service General Hospital, National Defense Medical Center, Taipei 11490, Taiwan; liyaofeng@ndmctsgh.edu.tw; 4 Department of Neurology, Tri-Service General Hospital, National Defense Medical Center, Taipei 11490, Taiwan; 5 Department of Biochemistry, National Defense Medical Center, Taipei 11490, Taiwan; emily7781@hotmail.com

**Keywords:** GrpE-like 2 homolog (*GRPEL2*), glioblastoma, oxygen consumption rate (OCR), autophagy, senescence

## Abstract

Malignant brain tumors are responsible for catastrophic morbidity and mortality globally. Among them, glioblastoma multiforme (GBM) bears the worst prognosis. The GrpE-like 2 homolog (*GRPEL2*) plays a crucial role in regulating mitochondrial protein import and redox homeostasis. However, the role of *GRPEL2* in human glioblastoma has yet to be clarified. In this study, we investigated the function of *GRPEL2* in glioma. Based on bioinformatics analyses from the Cancer Gene Atlas (TCGA) and the Chinese Glioma Genome Atlas (CGGA), we inferred that *GRPEL2* expression positively correlates with WHO tumor grade (*p* < 0.001), IDH mutation status (*p* < 0.001), oligodendroglial differentiation (*p* < 0.001), and overall survival (*p* < 0.001) in glioma datasets. Functional validation in LN229 and GBM8401 GBM cells showed that *GRPEL2* knockdown efficiently inhibited cellular proliferation. Moreover, GRPEL2 suppression induced cell cycle arrest at the sub-G1 phase. Furthermore, *GRPEL2* silencing decreased intracellular reactive oxygen species (ROS) without impending mitochondria membrane potential. The cellular oxidative respiration measured with a Seahorse XFp analyzer exhibited a reduction of the oxygen consumption rate (OCR) in GBM cells by *siGRPEL2*, which subsequently enhanced autophagy and senescence in glioblastoma cells. Taken together, GRPEL2 is a novel redox regulator of mitochondria bioenergetics and a potential target for treating GBM in the future.

## 1. Introduction

Over the past century, the treatment of malignant tumors of the brain has remained a critical challenge. However, the cure of malignancy has been elusive because it hardly achieves a complete excision of the intertwined tumor. Despite aggressive approaches, recurrence occurs in 90% of the patients after primary treatment. One cause of this poor consequence is the development of the multidrug-resistance (MDR) phenotypes, making prognosis frustrating [1,2,3]. Franco et al. discovered that high concentrations of ROS can damage mitochondrial-DNA (mt-DNA) and nuclear DNA, thereby altering the expression of oncogenes and tumor suppressor genes, which modifies the onset and progression of tumors developing MDR [4]. Newer combined pharmacologic strategies targeting multiple mitochondrial metabolic pathways were reported [2,5,6,7,8]. Recent evidence showed that the transcriptional down-regulation of *GRPEL2* at a microarray-based analysis brought about a treatment focus against lung cancer through metabolic reprogramming [9]. Glioma cells that rely on glycolytic metabolism readily adapt to bioenergetic stressors by engaging their mitochondrial pathway to survive and grow efficiently [10]. This observation suggests that the mitochondrial function plays an essential and promising role in the oncobiology of gliomas [1,11,12]. 

The specific role of GRPEL2 proteins is best known in the translocation of transit peptide-containing proteins from the inner membrane into the mitochondrial matrix in an ATP-dependent manner, acting as an essential component of the pre-sequence-associated motor (PAM) complex [2], controlling the nucleotide-dependent binding of the mitochondrial heat shock protein (mt-HSP) to substrate proteins and stimulating its own ATPase activity [13,14,15,16,17]. Its function of co-chaperon associated with an HSP of 70 kDa (HSP70) has been identified in vitro and validated in a structurally engineered crystal assay [18,19]. According to the study of Srivastava, we proposed that the human nucleotide exchange factor (NEF) paralog, GRPEL2, is responsible for tumorigenesis mediated by oxidative stress through mt-HSP machinery [6]. 

In the mitochondria, the protein-folding cycle is regulated by the NEF of HSP70, GrpE (GRPE), which facilitates the release of the folded proteins, and hence initiates the electron transport chain (ETC) activity [20], which exchanges ADP for ATP to maintain a high ATP/ADP ratio [21] and afford the enzymatic anaplerosis [16,22]. Recent advances reveal that the GRPEs serve distinct functions from the related HSPs. Our putative NEF ortholog, GRPEL2, modulates mitochondrial HSP70′s function in human GBM cell lines, suggesting that GRPEL2 has evolved as a possible anti-tumor hub emerging into modern cancer medicine [6,13]. 

This work aims to validate the function of *GRPEL2* to propose that the dysregulation of mitochondria in energy generation can lead to the apoptosis of glioblastoma cells. Besides, we envision the evolutionary incorporation of the human *GRPEL2* of mitochondria into the oncogenesis and bioenergetics of GBM. This finding shows that targeting the *GRPEL2* directly and indirectly alters the tumor mitochondria’s function, providing potential new avenues against GBM twilight [23]. 

## 2. Results

### 2.1. GRPEL2 Expression Correlated with Tumor Grade and Overall Survival in Glioma

**Figure 1:**
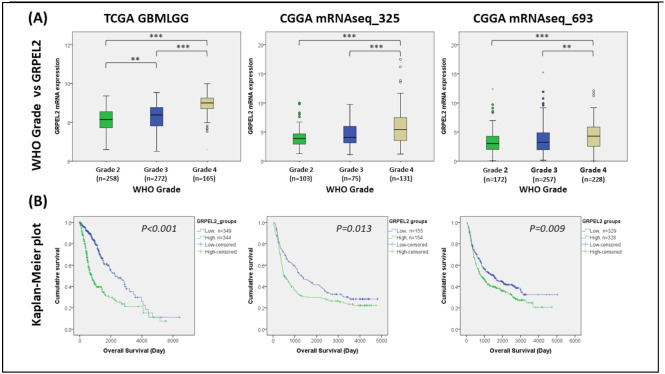
Elevated *GRPEL2* expression correlated with higher tumor grade and poorer prognosis in gliomas. (**A**) The boxplots showing *GRPEL2* gene expression correlate with the WHO grade of glioma patients in the TCGA and CGGA databases. ** *p* < 0.01, *** *p* < 0.001 (**B**) Kaplan–Meier graphs of *GRPEL2* mRNA expression level (high-expression versus low-expression groups) compared the survival probability from the TCGA and CGGA datasets. The plotted Kaplan–Meier curvatures used the log-rank test and the one-way ANOVA used for evaluating grade versus GRPEL2 (*p* < 0.001 for TCGA data; *p* = 0.013 for CGGA mRNAseq_325 and *p* = 0.009 for CGGA mRNAseq_693 datasets, respectively).

*GRPEL2* has been regarded as an auxiliary element in dysregulating mitochondria energy and protein import leading to apoptosis in gliomas [6,16]. To investigate the role of *GRPEL2* mRNA expression in glioma, we extracted the transcription data from The Cancer Genome Atlas (TCGA; UCSC Xena; https://xenabrowser.net/, accessed on 2 March 2021) and the Chinese Glioma Genome Atlas (CGGA; http://www.cgga.org.cn/, accessed on 2 March 2021). The multivariate statistical correlations were analyzed between *GREPL2* mRNA and clinical parameters in both databases. Upon subdividing the tumor groups in the TCGA dataset (GBMLGG) into Grade-II (n = 225), Grade-III (n = 272), and Grade-IV (n = 165), based on the WHO’s classification, we demonstrated that *GRPEL2* mRNA expression significantly differs among groups, and that more malignant (higher WHO grade) tumors had a higher expression level. This correlation was also demonstrated in the CGGA datasets (mRNAseq_325 and mRNAseq_693, *p* < 0.001, Figure 1A). We further divided the samples into high-*GRPEL2* (n = 344 at the TCGA dataset; n = 154 and 328 at the CGGA datasets) and low-*GRPEL2* (n = 349 at the TCGA dataset; n = 155 and 329 at the CGGA datasets) expression groups based on the median expression level. The Kaplan–Meier plots of three genome-wide clusters identically demonstrated that high-GRPEL2 expressions were significantly linked to a shorter survival time (*p* < 0.001 for TCGA; *p* = 0.013 and *p* = 0.009 for CGGA, respectively, Figure 1B). These results indicate that *GREPL2* acts as a strong indicator, impacting patients’ prognosis regarding tumor grade and overall survival. 

### 2.2. GRPEL2 Expression Correlated with Oligodendroglial Differentiation and IDH Wide-Type Phenotypes

**Figure 2:**
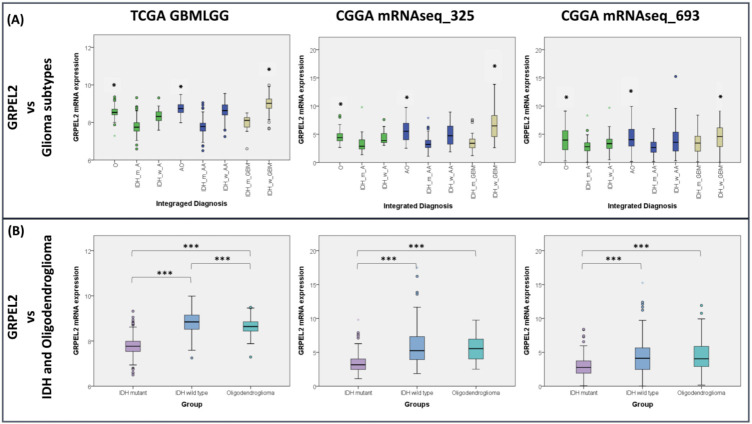
Elevated *GRPEL2* expression correlated with oligodendroglial differentiation and IDH status. (**A**) The boxplots showing *GRPEL2* mRNA expression correlate with phenotypes of oligodendroglioma (O), diffuse astrocytoma (A), anaplastic oligodendroglioma (AO), anaplastic astrocytoma (AA), and GBM in the TCGA and CGGA datasets. IDH status annotation: W (wide type) and M (mutant). * *p* < 0.05. (**B**) Graphs of *GRPEL2* mRNA expression level association with oligodendroglial differentiation and IDH status in the TCGA and CGGA datasets. *** *p* < 0.001.

Different studies propose that oligodendrocyte progenitor/precursor cells (OPCs) are the largest cellular differentiating population in the CNS in which GBM may originate [8] and progressively spread and tend to migrate along the fasciculus axons, where numerous OPCs are located [24]. Nonetheless, their function in glioma has not been as extensively studied as in the case of astrocytes. Utilizing multi-omic functional genomics data associated with glioma phenotypes showed that *GRPEL2* mRNA expression was exclusively correlated with oligodendroglial differentiation in the phenotypes of oligodendroglioma (O), anaplastic oligodendroglioma (AO), and GBM in all datasets (*p* < 0.05, Figure 2A). Isocitrate dehydrogenases (IDHs) are a group of enzymes that play a crucial role in the TCA cycle by catalyzing the oxidative decarboxylation of isocitrate to α-ketoglutarate (α-KG) and linking to the prognosis amongst anaplastic astrocytoma, GBM [2], and other forms of malignancy-associated metabolic reprogramming [23,25]. Our screening showed that the gliomas with wide-type IDH (wt-IDH), especially in the GBM subtype, exerted an elevated GRPEL2 expression in contrast to IDH mutant (mt-IDH) gliomas and oligodendrogliomas (*p* < 0.001, Figure 2B). Gilbert et al. have shown that wt-IDH GBMs up-regulate wt-IDH1, which fuels tumor growth and progression and displays therapy resistance [26]. Following studies on the knockdown of wt-IDH1 at in vitro models of depleted NADPH, deoxynucleotides and antioxidants hampered the ROS plasticity [11] and lipid biosynthesis [12] responsible for the maturation and myelination of OPCs, which later differentiate into oligodendrocytes. Our tentative connections between *GRPEL2*, oligodendroglial differentiation, and IDH status assumed that mitochondrial dysregulation plays a role in wt-IDH gliomas and oligodendroglial- enriched phenotypes.

### 2.3. GRPEL2-Stained Microarray Correlated with WHO Grade and Oligodendroglial Differentiation in Glioma Tissues

**Figure 3:**
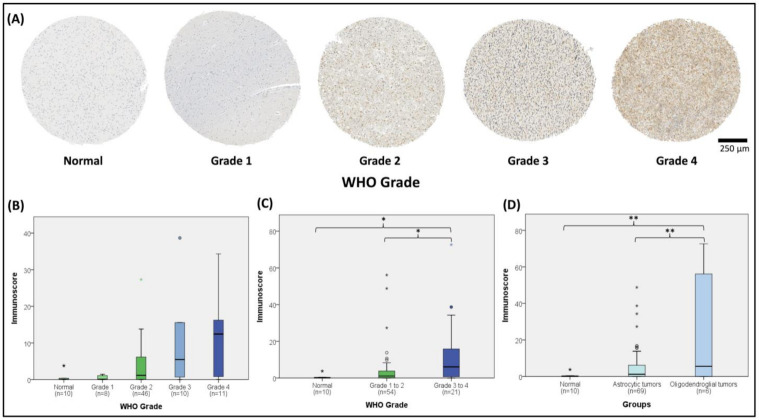
Immunohistochemical (IHC) anti-GRPEL2 staining of normal brain and gliomas with histological validation of TCGA/CGGA results. (**A**) The top panel displayed IHC-stained sections showing normal (non-neoplastic) brain, grade 1 (Gr-I), grade 2 (Gr-II), grade 3 (Gr-III) and grade 4 (Gr-IV) glioma tissues (from left to right). (**B**) showed elevated GRPEL2 protein staining (by immunoscore) significantly associated with WHO grades of gliomas (* *p* < 0.05), and the number for each grade was shown in parentheses. (**C**) showed elevated GRPEL2 protein staining (by immunoscore) correlated with higher-grade gliomas (grades 3 to 4) rather than lower-grade gliomas (grades 1 to 2) (* *p* < 0.05) (**D**) Elevated GRPEL2 protein staining (by immunoscore) was enhanced with oligodendroglial differentiation among histologically defined astrocytic tumor and gliomas with an oligodendroglial component (** *p* < 0.01). Note that GRPEL2 staining is greater at higher tumor grades. Scale bar in 250 μm.

To semi-quantitatively evaluate the GRPEL2 protein expression in human gliomas, we applied microarrayed tissue slides containing various WHO-grade gliomas for immuno-histochemical (IHC) staining. After validating the availability of the tissues, 95 samples (10 non-neoplastic brain tissues and 85 gliomas) were included in the analysis. Normal (non-neoplastic) brain tissue was stained negatively or was dimly faint for anti-GRPEL2 staining, whereas a greater GRPEL2 intensity was enhanced at higher-grade gliomas (Figure 3A). Further immunostaining confirmed that an elevated GRPEL2 staining (by immunoscore) significantly correlated with the WHO tumor grade (*p* < 0.05, Figure 3B) and that a greater GRPEL2 staining (by immunoscore) was associated with higher-grade gliomas (WHO 3 to 4, *p* < 0.05, Figure 3C). Similar results were also observed in tissues with oligodendroglial differentiation rather than an astrocytic counterpart among diverse gliomas, including pilocytic astrocytoma (PA), oligodendoglioma (OD), oligoastrocytoma (OA), anaplastic oligodendroglioma (AO), anaplastic oligoastrocytoma (AOA), anaplastic astrocytoma (AA), and glioblastoma multiforme (GBM) (*p* < 0.01, Figure 3D). These findings validated the finding that a higher *GRPEL2* is intensely expressed in more malignant gliomas and oligodendroglial differentiation. This finding was consistent with the correlative results from the TCGA and CGGA datasets.

### 2.4. GRPEL2 Expression Highly Correlated with TERT Rather Than ATRX Expression

Telomerase reverse transcriptase (TERT) is important for the biology of diffuse gliomas including GBM [27]. TERT promoter (TERT-*p*) mutations are selectively observed among 1p/19q-codeleted OD and wt-IDH GBM [25,28,29]. However, TERT transcripts range widely in various cancers, and their protein expression is highly correlated with *GREPL2* expression, in line with our analysis (r = 0.705, Table 1), although less correlations are observed for tumor grade (r = 0.456), ATRX expression (r = 0.454), patients’ age (r = 0.446), and IDH1 expression (r = 0.326). Our findings of *GRPEL2* expression linking to TERT expression in the wt-IDH gliomas other than oligodendroglial differentiation raise the possibility that the *TERT* mRNA could be regulated by a mechanism other than its promoter mutations, including the epigenetic alternative lengthening of telomeres (ALT) [25,30], the loss of alpha-thalassemia/mental retardation syndrome X-linked (ATRX) [31,32], and mitochondrial energetic dysregulation [28], while ATRX is less correlated with GREPL2 expression in our analysis (r = 0.454), rarely mutated in adult primary GBMs, but more common in younger adult patients with lower-grade gliomas [33]. This multivariate analysis indicated that the mitochondrial protein, GRPEL2, directly or indirectly impacted on the TERT protein for GBM progression. 

### 2.5. GRPEL2 Enhanced Expression in Glioma Cell Lines and Its Downregulation Decreased Cell Growth 

**Figure 4:**
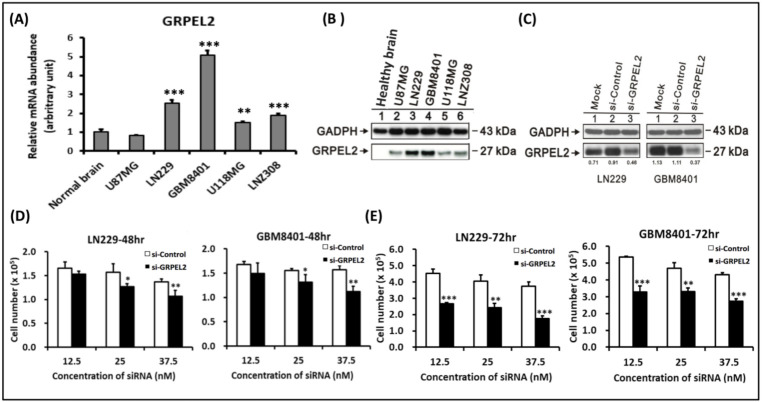
*GRPEL2* expression was enhanced in glioma cell lines and *GRPEL2* suppression inhibited cell survival. (**A**) Quantitative RT-PCR analysis of *GRPEL2* mRNA abundance in healthy brain tissue and the U87MG, the LN229, the GBM8401, the U118MG, and the LNZ308 glioma cells. ** *p* < 0.01, *** *p* < 0.001. (**B**) Western blotting analysis of GRPEL2 expression in healthy brain tissue and U87MG, LN229, GBM8401, U118MG, and LNZ308 glioma cells. (**C**) A representative Western blot validated the effect of *GRPEL2* knockdown in LN229 and GBM8401 glioma cells. (**D**,**E**) Quantitative validation of *siGRPEL2* to inhibit the cellular numbers (×10^5^) of LN229 and GBM8401 glioma cells at the concentration of 12.5 nM, 25 nM, and 37.5 nM siGRPEL2 in contrast to siControl treatment for 48 and 72 h. * *p* < 0.05, ** *p* < 0.01; *** *p* < 0.001.

To further investigate *GRPEL2* mRNA and protein expression in gliomas, we performed quantitative an RT-PCR and Western blotting of healthy brain tissue in contrast to the U87, LN229, GBM8401, U118MG, and LNZ308 glioma cell lines. We found that *GRPEL2* mRNA expressions and protein were significantly higher in all glioma cell lines, except for the U87 cells, than in healthy brain tissue (*p* < 0.01, Figure 4A,B). Next, we selected the top two cell lines, LN229 and GBM8401, with robust *GRPEL2* expression for suppression experiments. After the transfection of siGRPEL2 or siRNA (scramble siRNA) into the LN229 and GBM8401 glioma cells, the expression of the GRPEL2 protein was inhibited compared to the control (Figure 4C). The quantitative validation of GRPEL2 knockdown in both cell lines substantially suppressed cell growth at the 12.5 nM, 25 nM, and 37.5 nM concentrations of siGRPEL2 supplementation. The evaluation of cell growth after the GRPEL2 downregulation revealed that the number of glioma cells was significantly lower in siGRPEL2-expressing cells than in control. (*p* < 0.05, Figure 4D,E) More cell suppression was observed at a higher concentration of siGRPEL2. These findings indicated that inhibiting *GRPEL2* expression exerted anti-tumor growth in the LN229 and GBM8401 cell lines.

### 2.6. siGRPEL2-Induced Cell Growth Decrease Does Not Involve Apoptosis

**Figure 5:**
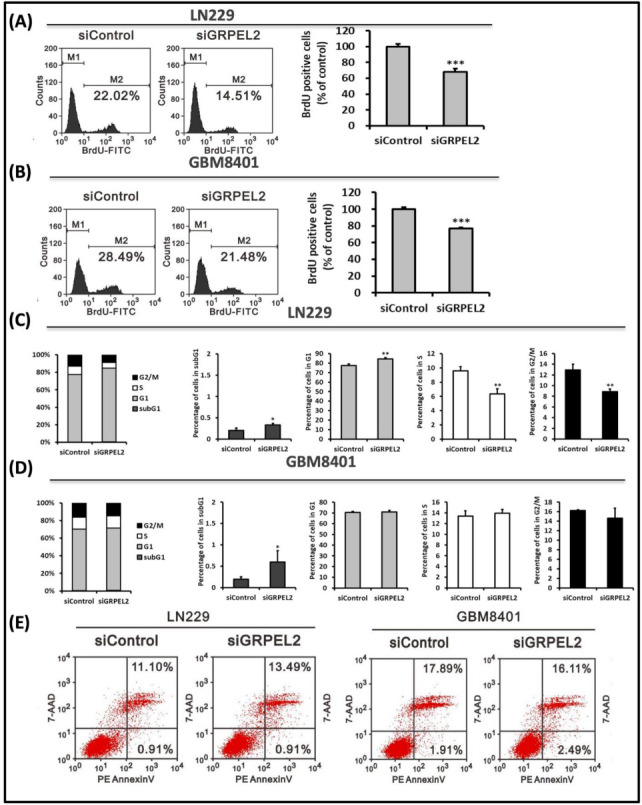
Effect of *siGRPEL2* on cellular proliferation and apoptosis of LN229 and GBM8401 cells. (**A**,**B**) FITC-labeled anti-BrdU staining of LN229 (**A**) and GM8401 (**B**) cells after incubation with siControl and *siGRPEL2* at 25 nM concentration analyzed by flow cytometry. M1 corresponds to the negative control and M2 to the positively stained cells (cell fractions expressed in percentage). Percentage of BrdU positive cells after treatment with siControl and *siGRPEL2* of LN229 cell lines. *** *p* < 0.001. (**C**,**D**) The cell cycle profiling of LN229 (**C**) and GBM8401 (**D**) cells was shown. Note that *GRPEL2* suppression increased cell fraction in the subG1 phase in both LN229 cells (**C**) and GBM8401 cells (**D**). * *p* < 0.05; ** *p* < 0.01. (**E**) Flowcytometric apoptosis assay of LN229 and GBM8401 cells treated with *siGRPEL2* after 7-AAD/PE Annexin V staining. The apoptotic cells (early and late stages) were detected by FCS and represented as a percentage. The data are representative of two independent experiments.

To confirm the role of *GRPEL2* on glioma cell growth, we examined LN229 and GBM8401 glioma cell growth by cell counting after *GRPEL2* silencing. Incubating *siGRPEL2* exhibited a drastic decrease of FITC-labeled BrdU-stained tumor cells compared to the control. LN229 cells survived 14.51% BrdU-positive cells (control: 22.02%), while GBM8401 cells also exerted the similar inhibition of cell proliferation (Figure 5A,B). Quantification analyses demonstrated the significant reduction of BrdU positive cells in both LN229 and GBM8401 clusters (inhibition fraction of 31.6% and 23%, respectively) (*p* < 0.001). Next, to investigate the impact of *GRPEL2* silencing on cell cycle in GBM cells, we analyzed the cell cycle profiling by flow cytometry. The result led to a slight increase in the numbers of cells at the subG1 phase in both cell-lines and a reciprocal decrease in cells at the S and G2/M phases in LN229 cells (Figure 5C,D). We further tested if *siGRPEL2* altered apoptosis in GBM cells. The 7-AAD/PE Annexin V assays revealed that *GRPEL2* knockdown did not appear to affect either early or late apoptosis in LN229 and GBM8401 cells. The net apoptotic fraction of LN229 cells after adding *siGRPEL2* increased from 12.01% to 14.40% (Figure 5E). The GRPEL2 knockdown did not show the prominent mutual alteration of canonical apoptotic proteins (PARP, BAX, and P53). In short, we speculated that the knockdown of *GRPEL2* retarded cell growth and inhibited cell proliferation via non-apoptotic signaling.

### 2.7. GRPEL2 Knockdown Decreased the Oxygen Consumption Rate (OCR) of Glioma Cells

**Figure 6:**
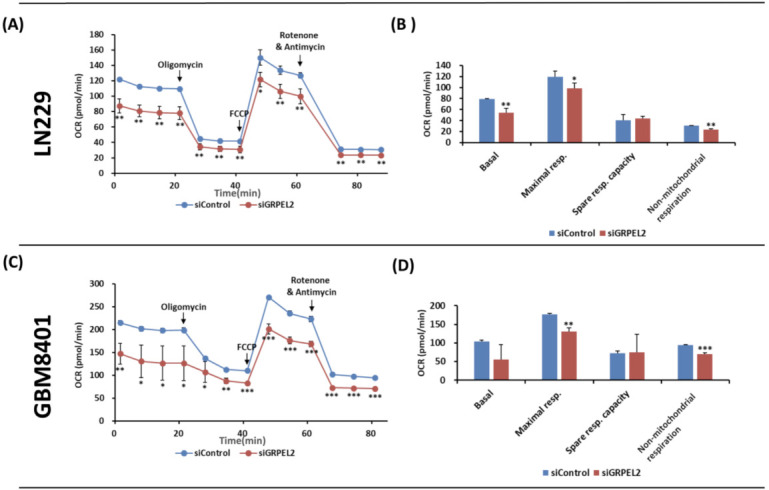
A Seahorse XF bioenergetic assay showed that GRPEL2 knockdown decreased OCR in LN229 and GBM8401 cells. (**A**,**C**) Oxygen consumption rate (OCR) of LN229 (**A**) and GBM8401 (**C**) cells treated with siControl and siGREPL2 in the Seahorse XFp analyzer. (**B**,**D**) Effect of GRPEL2 silencing on basal, maximal respiration, spare respiratory capacity, non-mitochondrial respiration coupling with cellular respiration of LN229 (**B**) and GBM8401 (**D**) cells. The graph A and C of OCR versus time (min) as shown is representative of four independent experiments in which three replicates of each assay were evaluated. * *p* < 0.05; ** *p* < 0.01; *** *p* < 0.001.

The Warburg hypothesis is featured with decreased ETC and ROS essentially decoupled from the normal, cellular energy-generating OXPHOS pathways [20,34], thus making glioma proliferation and development rely on cytosolic glycolysis by the lack of mitochondria-dependent glucose metabolism [2,16,23]. It is imperative to understand the underlying molecular interactions between the human GREPL2 and other stress-sensitive factors (ROS, transcription factors, IDH) that govern Krebs cycle’s overall redox regulatory processes. We had LN229 and GBM8401 cells measure the mitochondrial oxidative respiration using the Seahorse XF Extracellular Flux Analyzer. The results of OCR versus the time (minutes) of both cell lines were shown (Figure 6A,C). *GRPEL2* knockdown exhibited an obvious suppressive effect on the basal level, the maximal capacity, and non-mitochondrial respiration coupling with oxidative respiration in LN229 (*p* < 0.05, Figure 6B) and GBM8401 cells (*p* < 0.01, Figure 6D). These findings implied that the *GRPEL2* silencing of glioblastoma cells reduced mitochondrial OXPHOS, resulting in OCR decrease. The decreased non-mitochondrial respiration after *siGRPEL2* indicated intracellular ROS as another source of oxygen consumption. The anti-tumor effect comprising growth arrest and proliferative retardation can be explained by metabolic dysregulation. 

### 2.8. GRPEL2 Knockdown Reduced Intracellular ROS Activity without Disrupting the Mitochondrial Membrane Potential

**Figure 7:**
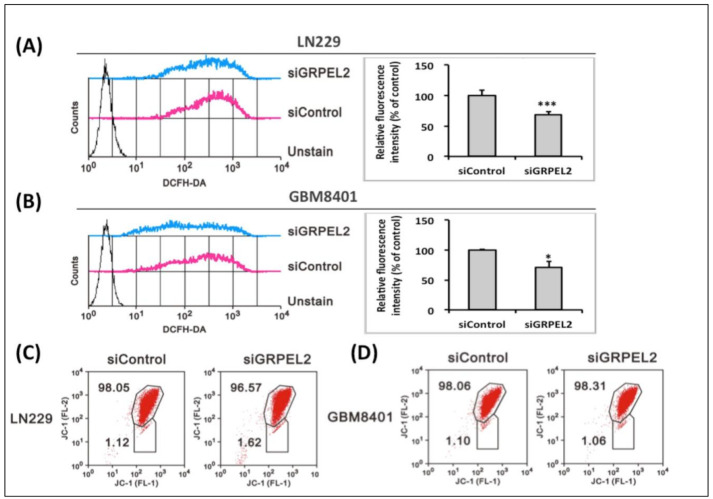
Effect of *GRPEL2* knockdown on intracellular ROS and mitochondrial membrane potential (mt-MP) in glioma cells. (**A**,**B**) The LN229 (**A**) and GBM8401 (**B**) cells were treated with 25 nM *siGRPEL2* for 24 h and stained with 2′-7′-dichlorodihydro fluorescein diacetate (DCFH-DA). The variation of the intracellular fluorescence intensity of LN229 and GBM8401cells was determined using flow cytometry (left panel). Relative fluorescence intensity analyses are shown on the right panel. * *p* < 0.05 *** *p* < 0.001. (**C**,**D**) Measure of mt-MP of LN229 (**C**) and GBM8401 (**D**) cells administrated with *siGRPEL2* (25 nM) for 48 h by JC-1 assay. The data are representative of two independent experiments.

Since OCR coupling with non-mitochondrial respiration was inhibited by *GRPEL2* knockdown, it is rational to demonstrate that human GRPEL2, for mt-HSP70, is a redox-sensitive protein forming in oxidative stress. An earlier in vitro study showed that GRPEL2 had a five-fold lower affinity for mt-HSP70 than GRPEL1, which is found more in physiological existence [6]. The current result confirmed that *GRPEL2* knockdown induced the ROS suppression in both LN229 and GBM8401 cells (*p* < 0.05, Figure 7A,B) but not P53-mediated apoptosis (Figure 5E). The reduced ROS effect is consistent with the decreased non-mitochondrial respiration after the *siGRPEL2* from the Seahorse XF bioenergetic assay (Figure 6B,D) The abrupt fluctuation of the ROS level triggered by *GRPEL2* down-regulation may lead to oxidative stress [16] and disrupt mitochondrial membrane potentials (mt-MP) [17]. Intriguingly, the mt-MP of LN229 and GBM8401 cells did not show an apparent shift after exposure to *GRPEL2* silencing (Figure 7C,D). This phenomenon implied that physiological GRPEL1 may be responsible for a major constitution for mt-MP maintenance [35], unlike pathological GRPEL2. These experiments showed that the knockdown of *GRPEL2*, provoking anti-tumor growth, inhibiting tumor proliferation, and mediating non-mitochondrial cytosolic glycolysis, could further manipulate intracellular ROS-induced GBM proliferation, eventually leading to the abrogated metabolic activity of GBM tumor cells.

### 2.9. siGRPEL2 Increased Autophagy and Senescence in Glioma Cells

**Figure 8:**
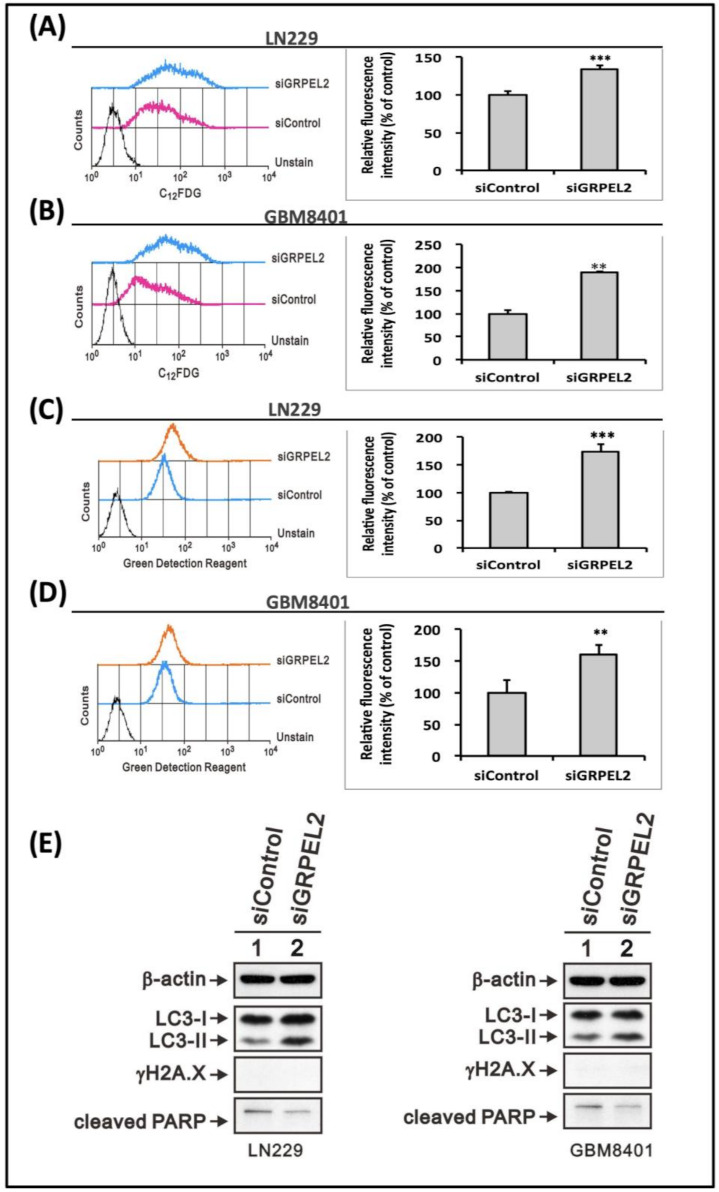
*GRPEL2* knockdown impeded cell viability via the enhanced autophagy and senescence of glioma cells. (**A**,**B**) Flow cytometry-based detection of senescence, LN229 (**A**), and GBM8401 (**B**) cells were exposed to *siGRPEL2* for 48 h incubated for C12FDG (5-dodecanoy lamino fluorescein di-β-d-galactopyrano side). Relative fluorescence intensity analyses are shown on the right panel. ** *p* < 0.01, *** *p* < 0.001. (**C**,**D**) Flow cytometry-based detection of autophagy, LN229 (**C**), and GBM8401 (**D**) cells were exposed to *siGRPEL2* for 48 h incubated for green detection reagent. Relative fluorescence intensity analyses are shown on right panel. ** *p* < 0.01, *** *p* < 0.001. (**E**) Western blotting of autophagy and apoptosis-related proteins in LN229 and GBM8401 cells treated with *siGRPEL2*, validating a prominent autophagic increase of the LC3-II to LC3-I ratio compared to the siControl. Note that γH2AX and cleaved PARP did not change. The images represent the results from three independent experiments. Β-actin served as a loading control.

The metabolic divergence between GBM and normal cells may provide novel therapeutic strategies to be exploited [1,34]. Increasing tumorigenicity correlates with a greater sensitivity to glycolytic inhibitors [7,25,28]. Indeed, autophagy, also known as type II programmed cell death, is now considered not only as a cell survival mechanism but also as a tumor suppressor hub [36] that induces the death of transformed cells under oxidative stress [37]. We used the flow cytometry to detect the senescence and autophagy of GBM cells treated with *siGRPEL2* at 25 μM, and the results showed that the inhibition of *GRPEL2* enhanced the senescent expression (*p* < 0.01, Figure 8A,B) and autophagic flux (*p* < 0.01, Figure 8C,D). *GRPEL2* inhibition can decrease survived glioblastoma cells through an oxidative stress-induced cascade and activated the senescence and autophagic flux restoration. These cellular modifications, associated with intracellular ROS depletion, are validated by the Western blot rather than canonical apoptosis (Figure 8E). These findings indicated that the down-regulation of *GRPEL2* inhibited GBM cells growth by modulating mitochondrial respiration through the proposed *GRPEL2* to mitochondrial communication, which cooperatively intensified autophagy and senescence.

## 3. Discussion

It has long been recognized that malignant tumors favor aerobic glycolysis to generate ATP and modified mitochondria-dependent apoptotic pathways [36], strongly suggesting the involvement of dysfunctional mitochondria in cancer pathophysiology [12]. This failure of tumor cells to progress to more efficient aerobic respiration for energy production was first described by the Nobel laureate Warburg [10], and subsequently termed the “Warburg hypothesis”. Later studies showed their reliance on persistent aerobic glycolysis as their main source of ATP production [20], as exhibited by new evidence from animal models [36] and glioma-derived cell cultures of in vivo human studies [37] presenting an inherent resistance to canonical p53-caspase apoptosis [2,25]. We re-examined Warburg’s observations in relation to the current concepts of cancer metabolism [38], intimately linked to alterations of mitochondrial protein import, energy disequilibrium, and redox homeostasis, and thus readily exploitable for cancer therapy in GBM patients. 

The over-expression of the GRPE protein is also widely reported in cancer cell lines, associating with aggressive growth and invasive properties [6,12,13,16,21,39]. As mentioned, the GRPE protein homolog takes a fundamental part in ATP-controlled cycles of substrate binding [15] and releases coupling with ETC by triggering protein activation [18,19]. Binding ATP/ADP initiates the chaperone function of mt-HSP70 [40] for its nucleotide-binding domain, where GRPEs exchange ADP for ATP, thereby regulating PAM activity [6,16]. In the present study, we investigated and tested the putative role of *GRPEL2* participating in gliomas exclusively from TCGA and CGGA screening. 

### 3.1. Impact of IDH/TERT Phenotype on GRPEL2 Expression 

TCGA has grouped glioblastomas relying on proteomic data into three classes: one showing epidermal growth factor receptor (EGFR) mutations or amplifications, the second having ligand-driven platelet-derived growth factor (PDGF) activation, and the third with a loss of RAS regulator, NF1 [41]. In recent years, numerous molecular alterations, including IDH mutations [38] and TERT-*p* [42,43], were identified and added to the primary and secondary glioblastomas’ core molecular landscape. Our preliminary bioinformatics from TCGA and CGGA mining unerringly exhibit that *GRPEL2* gene expression correlates with WHO tumor grades (*p* < 0.001), IDH mutation status (*p* < 0.001), and overall survival (*p* < 0.001), and highly correlated with *TERT* expression in gliomas. By screening our GBM genomic data, ODs and *IDH*^WT^ glioblastomas nearly exhibit a robust expression of *TERT*. An in-depth analysis of the TERT protein revealed that it contains a mitochondrial targeting sequence [44], and that it is regulated not only by phosphorylation [45], but also by changes in the intracellular redox homeostasis [46]. The mutual connection between TERT and ROS is also evident from the fact that a decrease in ROS triggered by *siGRPEL2* may entail a growth inhibition of GBM cells. These data demonstrate that mitochondrial telomerase activation may also interact with *GRPEL2*, affecting the redox homeostasis, and hence the ROS imbalance. Based on our experiments, it could be speculated that most of the redox-dependent functions as well as the regulation of the cellular bioenergetics can be assigned to the mitochondrial nucleotide switch.

We found *GRPEL2* expression to correlate with oligodendroglial differentiation in the TCGA and CGGA datasets, in line with other studies that had proposed OPCs as a potential niche where GBM may originate. Oligodendrocytes were presumed to have a tumor-supportive complicity with GBM, since eccentric migration tends to spread along the OPC-enriched fasciculi [8,24,47]. This evidence suggests that the GRPEL2 may recruit OPCs in the setting of glioma differentiation, which may be responsible for tumor recurrence and refractoriness to chemotherapy via the redox dysregulation of mitochondria. Further experiments are needed to validate this hypothesis.

### 3.2. siGRPEL2 Suppressed Non-Mitochondrial Respiration Alternating ROS

The utilization of LN229 and GBM8401 cells to proceed on *GRPEL2* silencing demonstrated a decreased cell growth and inhibited tumor proliferation by ROS-dependent manipulation. The Seahorse XFp respiration assay showed that a decreased *GRPEL2* expression did suppress the leakage of protons and ATP production in the mitochondria. Cellular proliferation is an energy-demand activity that is precisely controlled by checkpoints of the cell cycle [48]. Because of the TCA (Krebs) cycle and respiratory compromise, *GRPEL2* ablation triggered a metabolic overpowering of aerobic glycolysis [16], hence decreasing ROS stress [49]. It is well established that various therapeutic approaches based on ROS trigger cell death and reverse chemo-resistance in malignant tumors [20,25,36]. Exposing cancer cells to these chemo-therapeutic agents prompts an acute generation of intracellular ROS [2,17,48,50]. Such surge usually exhausts the cellular antioxidant capacity and brings ROS level beyond a threshold leading to apoptosis [1]. Franco et al. also discovered that high or low ROS concentrations can damage mitochondrial and nuclear DNA, therefore altering the expression of oncogenes and tumor suppressor genes, which modifies the onset and progression of tumors of diverse phenotypes [4]. Other nuclear-encoded mutations or a transcriptional up-regulation at the mt-DNA binding protein (ID2) [48] or IDH1/IDH2 [50] can also act like GRPEL2 to bring about the similar OXPHOS dysregulation through metabolic reprogramming and epigenetic deregulation of the associate gene expression. 

### 3.3. Role of siGRPEL2 in the Regulation of Mitochondrial ROS 

Knockdowning *GRPEL2* with *siGRPEL2* efficiently inhibits the cellular proliferation of both LN229 and GBM8401 cells. We speculated that *GRPEL2* down-regulation deterred energy production through aerobic respiration by manipulating ROS in the mitochondria. At physiological levels, ROS are non-lethal and act as intracellular messengers [36]. However, a prolonged excess of ROS in oxidative stress results in cellular damage and death by programmable apoptosis [25] or autophagy/senescence [37]. Moreover, increasing or decreasing ROS inhibited aerobic glycolysis [38] and impeded the exchange of ADP to ATP, hence deactivating ETC activity through the PAM complex [12]. Konovalova et al. had described that GRPEL2 is a redox-regulated protein in oxidative stress to form dimers through intermolecular disulfide bonds switched by the thiol Cys87 in the presence of ROS [16]. This suggests that the dimerization of GRPEL2 may activate the folding machinery responsible for protein import into the mitochondrial matrix or enhance the chaperone activity of mt-HSP70, thus protecting mitochondrial proteostasis in oxidative storm [1]. The reduced ROS effect is consistent with the decreased non-mitochondrial respiration after siGRPEL2 from the Seahorse XF bioenergetic assay. This indicated intracellular ROS as another source of oxygen consumption and the anti-tumor effect comprising growth arrest and proliferative retardation can be regulated by *GRPEL2* via the ROS level. 

### 3.4. siGRPEL2 Enhanced Autophagy and Senescence Expressions, Impacting on the Bioenergetics of Mitochondria 

Autophagy is regulated by numerous stresses, such as nutrient starvation, hypoxia, ATP/AMP ratio, intracellular ROS levels [37,51], bacteria and virus infection, or chemical drugs [2,25]. It has been demonstrated to be deregulated by ROS in particular neurodegenerative diseases [17] or several cancers [21]. In an indirect manner, ROS can modulate autophagy through AMP-activated protein kinase (AMPK), leading to the inhibition of the mammalian target of rapamycin 1 (mTORC1) and to autophagic induction [37,51]. This is then considered as a tumor suppressor mechanism by mainly preventing ROS accumulation through the elimination of damaged mitochondria comprising the major source of ROS. This selective autophagy, called mitophagy, is mediated by inducible putative kinase 1 (PINK1) [51]. Recent evidence showed that ROS can induce mutation in mitochondrial DNA and generate a feedback loop in which mutations in genes encoding PAM complexes of the ETC directly affect the efficiency of electron transport [23,25]. In reference to the nature of ROS behavior as a double-edged sword, even though several studies have documented the benefits of antioxidant drugs or the ROS-reducing strategy for cancer therapies, none has been supported by large-scale trials [2,17,29,34,50]. Poillet et al. proposed an important therapeutic approach to kill cancer cells by ROS-induced senescence dependent on the inactivation of autophagy-related gene-4 (ATG4), increasing LC3-associated autophagosomes and the ATM-mediated oxidation of AMP-activated protein kinase (AMPK), and inhibiting mTORC1 [51]. It has been clearly shown that the elimination of damaged mitochondria by senescent autophagy leads to decreased ROS production, thereby limiting the tumor-promoting effect [2,20]. Therefore, contrary to the initial “Warburg effect” dogma describing that cancer cells rely mainly on glycolysis during tumor growth, cancer cells are now described as presenting a decreased autophagy flux, a high oxidative phosphorylation, and a high ROS production. Nevertheless, the accumulation of ROS also increased the cytoprotective autophagy levels, leading to drug resistance and the survival of cancer cells [1,7,9,36]. In this particular strategy of metabolic intervention, depleting *GRPEL2* as the autophagy stimulators restored the tumoristatic sensitivity to the standard treatment [52] and decreased the ROS level, further inducing cell senescence [53]. This oncometabolic maneuver impedes the recycling of intracellular components to the mitochondrial importing machinery, thus decreasing the production of ATP and TCA cycle intermediates, which in turn leads to a decrease of ROS coupled with OXPHOS deactivation. Again, the bioenergetic impact sheds not only light, but also a torch blazing down on mitochondrial cancer medicine.

This study had several limitations. First, it was difficult to collect a large sample of non-tumor brain tissue and low-grade human gliomas to validate *GRPEL2* expression, although we performed a large-scale analysis of the TCGA and CGGA datasets to demonstrate that *GRPEL2* is a biomarker related to WHO pathological grading and survival outcome. We then confirmed the data analyzed by the three independent studies through wet lab approaches, such as qRT-PCR and Western blot. Second, the true significance of *GRPEL2* in the prediction of the survival prognosis in patients with GBM should be further investigated from human-derived tissues or in vivo studies. Third, more research efforts should be invested in associated mechanisms to explain the influences of *GRPEL2* on the PAM/ETC, OCR, ROS, and autophagy/senescence of GBM cells. Fourth, the most applicable strategy is to determine whether the GRPEL2 knockdown enhances the tumor sensitivity to the TMZ frontline chemotherapy, thus exerting cytotoxic synergism.

## 4. Conclusions

The modern trend in GBM research is heading toward molecular therapies focused at key points of tumor metabolism. Mitochondria reprogramming can contribute to cell death by influencing the redox and energetic equilibrium, coupling the disorderly ETC activity. Coining the anti-cancer drugs could trigger mitochondria to generate a ROS surge or waning that up-regulates apoptotic signaling. Our experiments showed that the inhibition of HSP-associated *GRPEL2* expression led to OXPHOS dysfunction, eliciting the intracellular ROS decrease, thereby retarding GBM cell proliferation. Additionally, by stimulating autophagy and senescence, *GRPEL2* ablation can be able to suppress the GBM growth.

In summary, we identified an oncometabolic connection between the human *GRPEL2* and glioblastoma cell lines. Our findings suggested *GRPEL2* as a potential regulator of mitochondria bioenergetics and possible target for GBM treatment.

## 5. Materials and Methods

### 5.1. Analysis of Data from the TCGA and CGGA Databases

To understand the role of GRPEL2 mRNA expression in glioma, we extracted the data from The Cancer Genome Atlas (TCGA; UCSC Xena) and the Chinese Glioma Genome Atlas (CGGA), after sorting and deleting the cases with missing values. The samples obtained from the TCGA_GBMLGG dataset (https://xenabrowser.net/, accessed on 2 March 2021) contained 662 cases, including 225 grade II, 272 grade III, and 165 grade IV glioma cases. Additionally, another two datasets including mRNAseq_325 and mRNAseq_693 obtained from the CGGA database (http://www.cgga.org.cn/, accessed on 2 March 2021). In the mRNAseq_325 dataset, there were 103 grade II, 75 grade III, and 131 grade IV cases with complete data. In the mRNAseq_693 dataset, there were 172 grade II, 257 grade III, and 228 grade IV cases with sufficient clinical information. Statistical comparisons of GRPEL2 mRNA expression among tumor grades were made using multiple t-tests and a one-way ANOVA. These cases were also divided into high and low GRPEL2 expression groups around the median GRPEL2 expression level. Kaplan–Meier plots were then constructed to compare the overall survival between the high and low GRPEL2 expression groups. 

### 5.2. Tissue Microarray Slide Preparation, Immunohistochemistry, and Scoring

The array of brain tumor tissue, clinical information, histology diagnosis, and pathology grade (GL1001a) was purchased from US Biomax Inc., https://www.biomax.us/ (accessed on 2 February 2021), for immunohistochemistry (IHC). The commercial Tissue Array contained 100 cases and validated the availability of total 85 tissues, including 10 normal brain tissues, 8 WHO grade 1 gliomas (astrocytoma, oligodendrogioma), 46 grade 2 gliomas (low-grade astroctyoma, low-grade oligodendrogliom), 10 grade 3 gliomas (oligodendroglioma, oligoastrocytoma), and 11 grade 4 gliomas (GBM) for IHC staining. The core measured 1 mm in diameter and had a 5-µm tissue thickness. Immunohistochemical staining was performed using the rabbit anti-GRPEL2 antibody (HPA023211, Atlas, Sweden), which ensured optimal results. Before staining, antigen retrieval was performed by heating the samples at 125 °C for 30 min in sodium citrate buffer (0.01 M sodium citrate, pH 6.2) using a pressure cooker. We switched off the cooker when it reached full pressure, and then waited until the pressure was released. Next, we took the retrieval slides and washed them 3 times for 5 min in a phosphate-buffered saline (PBS). Thereafter, the slides were stained following the manufacturer’s protocol, and the selected antibodies were evaluated by checking their binding to both positive and negative controls. Following the optimization of the retrieval and the antibody concentration, a robust staining of the positive but not the negative control was observed. The stained slides were then examined and scored using an automated semi-quantitative system, as in previous publications [54,55]. The slides were digitalized and exported as 10x tiff figures. The entire area was then automated and quantified using the Fiji software described in a previous work (REF: https://pubmed.ncbi.nlm.nih.gov/33797808/, accessed on 2 March 2021) and the macro also demonstrated in the (Supplementary File S1). In brief, the staining areas were scored from 0 to 3 as follows: 0 = negative, 1 = weakly positive, 2 = moderately positive, 3 = strongly positive. In addition, the percentage of tumor cells stained was estimated from 0% to 100%. Later, the percentage of cells at each intensity was multiplied by the corresponding intensity to generate an immunostaining score that ranged from 0 to 300. Finally, the immunostaining score of each sample was used for statistical analyses by performing a one-way ANOVA.

### 5.3. Statistical Analysis of the Association between GRPEL2 Expression and Other Factors

To determine whether GRPEL2 expression correlated with tumor grades, patients’ age, overall survival, and other possible prognostic factors (including TERT, ATRX, IDH1, TP53, EGFR, 1p19q status, MKI67, AXL, PDGFRA, CIC, NF1, and NF2) were determined. Firstly, the transcriptional data, tumor grades, patient’s age, and overall survival information were downloaded from the TCGA_GBMLGG dataset (https://xenabrowser.net/, accessed on 2 March 2021). Next, we used a Pearson’s correlation (r) to evaluate the relationship between these markers and determined their statistical importance. Values of *p <* 0.05 were considered significant.

### 5.4. Human Glioma Cell Lines and Lysate Preparation

The U87MG, U118MG, and LNZ308 human glioma cell lines were maintained in Dulbecco’s modified Eagle’s medium (DMEM) supplemented with 10% fetal bovine serum (FBS), 100 U/mL penicillin, and 100 mg/mL streptomycin. The LN229 and GBM8401 human glioma cell lines were maintained in Dulbecco’s modified Eagle’s medium (DMEM) supplemented with 2% fetal bovine serum (FBS), 100 U/mL penicillin, and 100 mg/mL streptomycin. The cultures were kept in an incubator under 5% CO_2_ at 37 °C. For the Western blot analysis of GRPEL2 expression, the cells were lysed in a RIPA lysis buffer, which contained 100 mM Tris-HCl, 150 mM NaCl, 0.1% SDS, and 1% Triton X-100. GAPDH served as an internal control. Normal brain cell lysate purchased from Novus Biologicals (NB820-59177, Littleton, CO, USA) also served as control.

### 5.5. Transfection of siGRPEL2 and siControl into Glioma Cell Lines and Cell Proliferation Assays 

GRPEL2 siRNA (siGRPEL2) and siControl were purchased from Dharmacon (product ID, M-016191-01-0010). For the cell proliferation assay, the cells were seeded into 12-well plates at a density of 2 × 10^4^ cells/well and incubated overnight at 37 °C. The next day, the cells were transfected with 25 and 37.5 nM siGRPEL2 or siControl (Dharmacon, CO, USA) using DharmaFECT 1 Transfection Reagent. To collect the cells, 200 µL 10% FBS DMEM and 100 µL of 0.05% trypsin were added to each well. Cell counting was tested 48 and 72 h after transfection. After preparing a mixture of 10 µL of each cell line and 10 µL trypan blue, the cells were counted, excluding the dye, using a TC20™ Automated Cell Counter (Bio-Rad, Hercules, CA, USA). The cell counting assays were replicated three times with each cell line. The FITC-labeled anti-BrdU staining of LN229 and GM8401 cells after incubation with siControl and siGRPEL2 at a 25 nM concentration was analyzed by flow cytometry. The cells were transfected with siRNA and then processed with the FITC-BrdU Flow Kits according to the manufacturer’s instructions (BD Biosciences).

### 5.6. RNA Isolation and Real-Time Reverse Transcription-PCR (RT-PCR)

Total RNA was extracted using TRIzol™ Reagent (Thermo Fisher Scientific, Waltham, MA, USA) and reverse-transcribed to single-stranded cDNA withIQ2 MMLV RT-Script (Bio-Genesis Technologies, Taipei, Taiwan). For qRT-PCR, the PCR reactions were carried out using a StepOne™ Real-Time PCR System (Thermo Fisher Scientific, USA) with Fast Plus EvaGreen qPCR Master Mix (Biotium, Fremont, CA, USA). Thermocycling was performed with 0.25 µM of each primer (PrimerBank) and 2.5 µL of the cDNA diluent. The PCR protocol entailed a denaturation for 2 min at 95 °C followed by 40 cycles of a touch-down PCR protocol (5 s at 95 °C and 30 s annealing at 60 °C). The primers used were as follows: for GRPEL2, 5′-GAGCCAAAACACCAAGCCTTA-3’(forward) and 5′-GGAGTTTAATGCT GATGGACCTT-3′(reverse); for GAPDH, 5’-GCACCGTCAAGGCTGAGAAC-3’(forward), and 5’-ATGGTGGTGAAGACGCCAGT-3’ (reverse).

### 5.7. Western Blot Analysis (Lysate Preparation Was Referred to in 4.4)

The glioma cell lines (including LN229, GBM8401, U87MG, U118MG, and LNZ308) were washed twice with PBS and lysed in a RIPA buffer (100 mM Tris-HCl in pH 8.0, 0.1% SDS, 150 mM NaCl, and 1% Triton 100). The protein lysates (20–40 µg, depending on the concentration) were separated by 10% SDS-PAGE and analyzed by immunoblotting with antibodies against polyclonal rabbit anti-human GRPEL2 (HPA023211, Atlas, Sweden), anti-LC3 (#2775, Cell Signaling Technology, Danvers, MA, USA), anti-cleaved PARP (#9546, Cell Signaling Technology, USA), and monoclonal mouse anti-GAPDH (sc-47724, Santa Cruz, Dallas, TX, USA) antibodies. 

### 5.8. Cell Cycle Assessment

LN229 and GBM8401 cells were transfected with 25 nM siGRPEL2 for 72 h, harvested, fixed with 70% ethanol, briefly washed with PBS/1% FBS, and then incubated with 10 mg/mL RNAse A and 50 mg/mL propidium iodide in PBS plus 1% Tween 20 for 30 min at 37 °C in the dark. A flow cytometric analysis was performed using a FACS Calibur flow cytometer (BD Biosciences, Franklin Lakes, NJ, USA). The cell fractions in the respective cell cycle phases were calculated using the Cell Quest Pro software (BD Biosciences, Franklin Lakes, NJ, USA).

### 5.9. Measurement of Oxygen Consumption Rate (OCR)

Cellular oxidative respiration was measured with the Seahorse XF bioenergetic assay using the Seahorse Cell Mito Stress Test Kit (Seahorse Bioscience, North Billerica, MA, USA). In brief, LN229 and GBM8401 glioma cells were plated in a 10-cm culture dish and incubated overnight, after which they were transfected with siGRPEL2 or siControl as described above. After 48 h of transfection, LN229 and GBM8401 cells were seeded onto an XFp microplate in DMEM supplemented with 2% FBS. They were then incubated for 1 day, after which the medium was replaced with sodium bicarbonate-free DMEM supplemented with 2% FBS. The OCR was measured at a steady state, after which the machine sequentially added oligomycin (1 μM), carbonyl cyanide 4- [trifluoro methoxy] phenylhydrazone (FCCP; 0.5 μM), and a mixture of rotenone (0.5 μM) and myxo thiazol (1 μM) into the wells to obtain the maximal and non-mitochondrial respiration rates according to the manufacturer’s instructions. 

### 5.10. Intracellular ROS, Senescence, and Autophagy Determination 

2’-7’-dichlorodihydrofluorescein-diacetate (DCFH-DA) penetrates cells and becomes hydrolyzed to non-fluorescent dichlorodihydrofluorescein (DCFH). DCFH reacts with ROS to form the highly fluorescent dichlorofluorescein (DCF), which can be measured by a flow cytometry. Briefly, LN229 and GBM8401 glioma cells were plated in 6-well plates at a density of 1 × 10^5^ cells/well and incubated overnight, after which they were transfected with siGRPEL2 or siControl as described above. After incubation for an additional 24 h, the cells were incubated with 10 μM DCFH-DA (Sigma, St. Louis, MO, USA) for 10 min at 37 °C in the dark and washed twice with DPBS. ROS levels were analyzed using a flow cytometer (FACSCalibur, BD Biosciences, San Diego, CA, USA). For the flow-cytometry-based detection of senescence, the cells were exposed to siGRPEL2 for 48 h. Thereafter, 33 µM C12FDG (Molecular Probes, Eugene, OR, USA) was added and incubated for 60 min at 37 °C. The medium was removed, and the cells were washed with PBS, trypsinized, and resuspended in PBS. Senescence was measured at the BD FACSCalibur flow cytometer. For the flow cytometry–based detection of autophagy, the cells were exposed to siGRPEL2 for 48 h. Thereafter, 33 µNM green detection reagent in the fluorescence of tagged LC3 (Molecular Probes, USA) was added and incubated for 60 min at 37 °C. The medium was removed, and the cells were washed with PBS, trypsinized, and resuspended in PBS. Autophagy was measured at the BD FACSCalibur flow cytometer. 

### 5.11. Measurement of Mitochondrial Membrane Potential

Mitochondrial potential was measured using a BD™ MitoScreen Flow Cytometry Mitochondrial Membrane Potential Detection Kit (BD Biosciences) according to the manufacturer’s protocol. Briefly, the cells were exposed to siGRPEL2 for 48 h in 6-well plates. The cells were then trypsinized and pelleted by centrifugation at 1000 rpm, after which the cells were resuspended in PBS and counted, which confirmed that there were fewer than 1 × 10^6^ cells per mL. The cells were then stained with JC-1 dye (5,5′,6,6′- tetrachloro-1,1′,3,3′-tetraethylbenzimi-dazolylcarbocyanine iodide) for 10–15 min at 37 °C in a CO_2_ incubator. The fluorescence intensity of the JC-1 was evaluated by flow cytometry. The excitation wavelength was 488 nm, while emission wavelengths of 530 nm (FL1-H channel) and 580 nm (FL2-H channel) were used to detect the JC-1 monomer and aggregates, respectively.

### 5.12. Flow Cytometry Analysis

Annexin V-PE/7-AAD kits (Becton Dickinson, Franklin Lakes, NJ, USA) were used to measure the apoptosis of glioma cells. Following the manufacturer’s instructions, the cells were digested and collected, washed for 2 times, and stained with Annexin V-PE/7-AAD for 15 min in the dark. Apoptosis was analyzed by the FACS Calibur flow cytometer (Becton Dickinson, Franklin Lakes, NJ, USA). Negative PE Annexin V and 7-AAD indicate viability; A positive PE Annexin V and a negative 7-AAD indicate early apoptosis; Positive PE Annexin V and 7-AAD indicate late apoptosis or dead. Early apoptosis and late apoptosis were summed, and the total apoptosis rate was calculated.

## Figures and Tables

**Table 1:** Correlation of risk factors associated with *GRPEL2* expression in gliomas.

	Multivariate Analysis		
Parameters	Numbers	Pearson Correlation (r)	Significance (2-Tailed)
TERT expression	688	0.705	1.52 × 10^−104^ *
Tumor grade	686	0.456	1.30 × 10^−36^ *
ATRX expression	688	0.454	3.24 × 10^−36^ *
Patients age	686	0.446	6.59 × 10^−35^ *
IDH1 expression	688	0.326	1.72 × 10^−18^ *
TP53 expression	688	0.270	5.98 × 10^−13^ *
EGFR expression	688	0.270	5.92 × 10^−13^ *
1p19q status	688	0.246	6.30 × 10^−11^ *
MKI67 expression	688	0.230	1.10 × 10^−09^ *
OS time	685	−0.198	1.78 × 10^−07^ *
AXL expression	688	−0.231	9.36 × 10^−10^ *
PDGFRA expression	688	−0.239	2.33 × 10^−10^ *
CIC expression	688	−0.357	4.24 × 10^−22^ *
NF1 expression	688	−0.318	1.27 × 10^−17^ *
NF2 expression	688	−0.409	4.62 × 10^−29^ *

(r) Differences were analyzed using the Pearson correlation and Cox proportion hazards regression method. * indicates the statistical significance, *p* < 0.025, in the 2-tailed test. Abbreviations: TERT: telomerase reverse transcriptase, ATRX: alpha-thalassemia/mental retardation syndrome X-linked, IDH: isocitrate dehydrogenase, EGFR: epidermal growth factor receptor, MKI67: marker of proliferation Ki-67, AXL: AXL receptor tyrosine kinase, PDGFRA: platelet-derived growth factor receptor alpha, CIC: capicua transcriptional repressor, NF: neurofibromatosis.

## Data Availability

The data presented in this study are available in the Supplementary Material.

## Supplementary Materials

The following is available online at https://www.mdpi.com/article/10.3390/ijms222312705/s1.

## Abbreviations

CGGA	Chinese Glioma Genome Atlas
GBM	Glioblastoma multiforme
GRPEL2	GrpE protein homolog-2
IHC	Immunohistochemistry
NEF	Nucleotide exchange factor
PAM	Pre-sequence-Associated Motor
ROS	Reactive Oxygen Species
TCGA	The Cancer Gene Atlas
WHO	World Health Organization
