# Supplementary material for: GRPEL2 Knockdown Exerts Redox Regulation in Glioblastoma
Source: Int J Mol Sci. 2021 Nov 24;22(23):12705. doi: 10.3390/ijms222312705 (PMC8657957; doi:10.3390/ijms222312705)
Supplement: Supplementary file 1 [file ijms-22-12705-s001.zip › ijms-1442421 - supplementary-for publish.pdf]

International Journal of *Molecular Sciences*

MDPI

Supplementary Materials

GRPEL2 Knockdown Exerts Redox Regulation in Glioblastoma

Chi-Tun Tang [1,2], Yao-Feng Li [3], Chung-Hsing Chou [2,4], Li-Chun Huang [5], Shih-Ming Huang [2,5], Dueng-Yuan Hueng [1,2,5], Chia-Kuang Tsai [2,4,*] and Yuan-Hao Chen [1,2,*]

[1] Department of Neurological Surgery, Tri-Service General Hospital, National Defense Medical Center,
Taipei 11490, Taiwan; 803010304@mail.ndmctsgh.edu.tw (C.-T.T.); hondy2195@yahoo.com.tw (D.-Y.H.)
[2] Graduate Institute of Medical Sciences, National Defense Medical Center, Taipei 11490, Taiwan; choutpe@yahoo.com.tw (C.-H.C.); shihming7102@gmail.com (S.-M.H.)
[3] Department of Pathology, Tri-Service General Hospital, National Defense Medical Center, Taipei 11490, Taiwan; liyaofeng@ndmctsgh.edu.tw
[4] Department of Neurology, Tri-Service General Hospital, National Defense Medical Center, Taipei 11490, Taiwan
[5] Department of Biochemistry, National Defense Medical Center, Taipei 11490, Taiwan; emily7781@hotmail.com
* Correspondence: jiakuang@office365.ndmctsgh.edu.tw (C.-K.T.); chenyh178@gmail.com (Y.-H.C.)

ImageJ macro for quantifying GRPE2L staining

```
macro "3 level Dab" {
 dir1 = getDirectory("Input Folder images");
 dir2 = getDirectory("Choose a folder to save to");
  list = getFileList(dir1);
  setBatchMode(true);
      for (i=0; i<list.length; i++) {
 showProgress(i+1, list.length);
 filename = dir1 + list[i];
 open(filename);
 Imagename = File.nameWithoutExtension;

# For strong staining 3+ area
 Name=getTitle();
 run("8-bit");
 run("Duplicate...", "title=1st");
 rename(Name+"-1st");
 run("Threshold...");
 setThreshold(0, 50);
 setOption("BlackBackground", false);
 run("Convert to Mask");
 run("Analyze Particles...", " show=Masks
  summarize");
```

```
# For weak staining 1+ area
selectWindow(Name);
rename(Name+"-3rd");
run("8-bit");
run("Threshold...");
setThreshold(101, 150);
setOption("BlackBackground", false);
run("Convert to Mask");
run("Analyze Particles...", "   show=Masks summarize");
selectWindow(Name);

# For whole tissue area, including 0, 1+, 2+, 3+
run("Duplicate...", "title=4th");
rename(Name+"-4th");
run("8-bit");
run("Threshold...");
setThreshold(0, 245);
setOption("BlackBackground", false); run("Convert to
Mask");
run("Fill Holes");
run("Analyze Particles...", "size=5000-Infinity
show=Masks include summarize");
```

# For modearte staining 2+ area

```
selectWindow(Name);
run("Duplicate...", "title=2nd");
rename(Name+"-2nd");
run("8-bit");
run("Threshold...");
setThreshold(51, 100);
setOption("BlackBackground", false);
run("Convert to Mask");
run("Analyze Particles...", " show=Masks summarize");
run("Close All"); }
selectWindow("Summary");
saveAs("Results", dir2+"Summary.csv");
exit("DAB measured in "+i+" images"); }
```
